# Infection with multidrug-resistant *Campylobacter coli* mimicking recurrence of carcinoid syndrome: a case report of a neuroendocrine tumor patient with repeated diarrhea

**DOI:** 10.1186/s12879-016-1743-4

**Published:** 2016-08-12

**Authors:** Heimo Lagler, Barbara Kiesewetter, Markus Raderer

**Affiliations:** 1 Department of Medicine I, Division of Infectious Diseases and Tropical Medicine, Medical University of Vienna, Währinger Gürtel 18-20, 1090 Vienna, Austria; 2 Department of Medicine 1, Division of Oncology, Medical University of Vienna, Währinger Gürtel 18-20, 1090 Vienna, Austria

**Keywords:** Campylobacteriosis, Carcinoid syndrome, Neuroendocrine Tumor, Chronic Diarrhea

## Abstract

**Background:**

Campylobacteriosis caused by Gram-negative bacteria of the genus *Campylobacter* (mainly *C. jejuni* and *C. coli*) is one of the most common gastrointestinal zoonotic infections with increased incidence in humans worldwide. The typical symptoms are severe abdominal cramps, diarrhea and sometimes fever. The clinical course of *Campylobacter* infection is mainly mild and after one week self-limiting, but can take several weeks in some rare cases. However, patients with neuroendocrine tumors in the gastrointestinal tract, a neoplasm of enterochromaffin/neuroendocrine cell origin, can develop severe diarrhea during progression of tumor growth caused by hormonal excess due to the tumor. Both diseases have very similar clinical symptoms and this case report elaborates the differences. So far it is known in the literature that the clinical symptoms of campylobacteriosis can mimic appendicitis or acute colitis of inflammatory bowel disease but a mimicking of recurrence of carcinoid syndrome in a patient with neuroendocrine tumor is not reported.

**Case presentation:**

A 72-year-old man with already diagnosed and treated metastatic neuroendocrine tumor of the terminal ileum (G1 rated, Ki-67 index 1 %) was again suffering from increasing diarrhea, abdominal cramps and weight lost. These symptoms were similar to the initial symptoms due to the tumor which improved at the time after total resection of the primary in the terminal ileum and regular therapy with long-acting release depot octreotide intramuscularly. As progression/tachyphylaxis in symptomatic patients with carcinoid syndrome undergoing therapy, reassessment of disease and analysis of tumor markers was initiated, and the interval of intramuscular injections was shortened. Radiological findings and tumor marker levels disclosed no evidence of neuroendocrine tumor progression and the symptoms continued. After 4 weeks with symptoms the patient developed additionally fever. Due to impaired renal function and elevated signs of systemic inflammation fluid replacement and empiric antimicrobial therapy were started. At this time-point the first stool cultures were taken which disclosed an infection with *C. coli*. The empiric antimicrobial therapy was stopped after five days because of multidrug-resistant isolated strain. During the ongoing symptomatic therapy the patient becomes gradually symptom-free 6 weeks later, resulting a total duration of symptoms caused by campylobacteriosis of 13 weeks.

**Conclusion:**

This case of infection with *C. coli* mimicking recurrence of carcinoid syndrome suggests that assessment for bacterial gastrointestinal infections should be taken into account also in patients with neuroendocrine tumors who present worsening of their symptoms in spite of initially successful management. The duration of symptoms caused by campylobacteriosis were significantly extended which might be due to gastroenteric dysfunctions/mucosal changes caused by the carcinoid syndrome in this patient.

## Background

Campylobacteriosis caused by Gram-negative bacteria of the genus *Campylobacter* (mainly *C. jejuni and C. coli*) is one of the most common gastrointestinal zoonotic infection with increased incidence in humans in both developed and developing countries worldwide [1, 2]. The source of infection is mainly contaminated food (particular undercooked poultry products, unpasteurized milk or water) or a close contact to animals [2–4]. After a short incubation period of few days, the typical clinical symptoms with abrupt onset are severe abdominal cramps associated with watery or bloody diarrhea with 8–10 bowel movements per day, weight loss and fever [5]. Usually, the infection with *Campylobacter* is self-limiting without any antimicrobial treatment and the clinical symptoms resolved after a median duration of 6 days [3]. During the time of symptoms supportive therapy like the maintenance of proper hydration and correction of electrolyte abnormalities are sufficient. The bacterial excretion takes 1–3 weeks, but can take several weeks in some cases, chronic carriers are rarely found and are mainly associated with immune deficiency [6, 7].

Patients with neuroendocrine tumors (NETs) in the gastrointestinal tract, a neoplasm of enterochromaffin/neuroendocrine cell origin, develop during progression of tumor growth typically diarrhea and weight lost caused by hormonal excess due to the tumor [8]. The clinical symptoms of the first presentation or progression of a NET of the gastrointestinal tract are with abdominal cramps, diarrhea and weight lost, similar to a bacterial gastroenteritis like campylobacteriosis without fever. Carcinoid syndrome is usually characterised by watery diarrhea without relevant amounts of mucus and blood, which differs sometimes from diarrhea caused by bacterial pathogens like *Campylobacter* where blood and mucus can be found in the diarrheal stool. It has already been described that the clinical symptoms of campylobacteriosis can mimic other diseases including acute appendicitis [9–11] or acute colitis of inflammatory bowel disease [12]. However, in the literature no case of campylobacteriosis mimicking clinical symptoms of a progression of a metastatic neuroendocrine tumor of the gastrointestinal tract is described.

## Case presentation

A 72-year-old man with already diagnosed metastatic NET and suffering from abdominal cramps, diarrhea and weight loss since 3 weeks was referred at our outpatient department. The NET had been located in the terminal ileum and had been diagnosed from biopsy of multiple liver metastases evidenced on CT-scanning nineteen months ago. Before undergoing clinical work-up and diagnosing the NET, the patient related an almost 12 month history of increasing diarrhea (up to 15 bowel movements per day) with weight loss of 11 kg. The tumor was rated G1 with a Ki-67-labeling index of 1 % [13], and the primary in the terminal ileum was resected. At diagnosis, the serum level of tumor marker chromogranin A was elevated at 1080 ng/ml (normal range <100 ng/ml), and the urinary 5-hydroxyindoleacetic acid (5-HIAA) was 29 mg/24 h (normal range 2 – 9 mg/24 h). After perioperative prevention with short-acting somatostatin (SST)-analogue octreotide subcutaneously, the patient was put on long-acting release depot octreotide LAR (OCT-LAR) 30 mg intramuscularly every 28 days, resulting in decrease of stool frequency to 2–3 bowel movements/day and weight gain by 7 kg.

Nineteen months after initiation of OCT-LAR, however, the patient again complained of severe diarrhea with up to 10 bowel movements with weight loss of 4 kg over 3 weeks. As progression/tachyphylaxis in symptomatic patients with carcinoid syndrome undergoing therapy with SST-analogues, reassessment of disease status performing a PET/CT with a SST receptor PET tracer [^68^Ga-DOTA,1-Nal^3^]-octreotide (^68^Ga-DOTANOC) and analysis of tumor markers was initiated, and the interval of OCT-LAR intramuscular injections was shortened to 21 days. However, radiological findings disclosed no evidence of NET progression. Also the regularly monitored tumor marker levels of chromogranin A and serotonin had been stable over the last months and were not indicative of progression (chromogranin A: 215 ng/ml; normal range <100 ng/ml and serotonin: 394 ng/ml; normal range 40–400 ng/ml). After 4 weeks with symptoms the patient developed additional fever up to 39 °C and night sweats for 2 days. Because of elevated signs of systemic inflammation (CRP: 20 mg/dl; normal range <0.5 mg/dl) and acute renal failure (serum creatinine: 6.7 mg/dl; normal rage 0.7–1.2 mg/dl) the outpatients was hospitalized and supportive therapy with intravenous hydration and maintenance of electrolyte balance plus empiric antimicrobial therapy with ciprofloxacin and metronidazole was started. At this time-point the first stool cultures were taken to identify microorganisms causing diarrhea like *Salmonella*, *Campylobacter*, *Shigella* and *Yersinia*. Stool samples were plated onto MacConkey agar (Oxoid, UK), Hektoen agar (bioMérieux, France), Cefsulodin-Irgasan-Novobiocin (CIN) agar (Becton Dickinson, Germany) and Campylosel agar (bioMérieux, France) as well as inoculated in Selenite F broth. All samples were incubated at 35–37 °C for 24 h. Suspicious colonies growing on Campylosel agar (a selective medium for the isolation of *Campylobacter*) were identified using matrix-assisted laser desorption ionization time-of-flight mass spectrometry (MALDI-TOF, Bruker, Germany) as described by the manufacturer. The result obtained by MALDI-TOF was consistent with the presence of *C. coli*. The following antimicrobial susceptibility testing according to European Committee on Antimicrobial Susceptibility Testing (EUCAST) disclosed a multidrug-resistant (MDR) *C. coli* (resistant to all 3 antimicrobial classes, the macrolides, fluoroquinolones and tetracyclines). Blood cultures and stool testing for *Clostridium difficile* toxins were negative. In view of limited therapeutic options due to the MDR and the usually self-limiting nature of campylobacteriosis, the antimicrobial therapy was stopped after 5 days and a wait and see approach was chosen. The patient became symptom-free with normalization of serial stool cultures 6 weeks later, resulting in a total duration of symptoms of 13 weeks.

## Conclusion

The clinical symptoms of Campylobacteriosis can mimic not only appendicitis or acute colitis of inflammatory bowel disease but also recurrence of carcinoid syndrome in a patient with NET. In addition to the four most frequent and harmful bacterial enteric pathogens *Salmonella*, *Campylobacter*, *Shigella* and *Yersinia* tested by a routine stool culture, also other bacteria like *Clostridium difficile* and enteropathogenic *Vibrio* species or *Escherichia coli* strains could cause similar clinical symptoms. However, if bacterial stool examinations are negative, additional virological analyses for example for detecting cytomegaly virus (CMV) and parasitological analysis for example for detecting *Entamoeba histolytica* or *Giardia lamblia* in the stool should be considered.

Interestingly this patient developed mainly watery diarrhea with a little mucus but without blood. However, the clinical symptoms with abdominal cramps caused by *C. coli* were very similar to the symptoms caused by the NET and therefore the infection was remaining undiagnosed for weeks. The duration of 13 weeks of symptoms caused by *Campylobacter* was remarkably extended, which might be due to gastroenteric dysfunctions or mucosal changes caused by the carcinoid syndrome.

This case of infection with *C. coli* mimicking recurrence of carcinoid syndrome suggests that assessment for bacterial gastrointestinal infections should be taken into account also in patients with NETs who present worsening of their symptoms in spite of initially successful management.

## Abbreviations

5-HIAA, 5-hydroxyindoleacetic acid; ^68^Ga-DOTANOC, [^68^Ga-DOTA,1-Nal^3^]-octreotide; CIN, cefsulodin-irgasan-novobiocin; CMV, cytomegaly virus; CRP, C-reactive protein; CT, computed tomography; EUCAST, European Committee on Antimicrobial Susceptibility Testing; MALDI-TOF, matrix-assisted laser desorption ionization time-of-flight mass spectrometry; MDR, multidrug-resistant; NET, neuroendocrine tumor; OCT-LAR, long-acting release octreotide; PET, positron emission tomography; SST, short-acting somatostatin

## References

[1] Kaakoush NO, Castano-Rodriguez N, Mitchell HM, Man SM (2015). Global Epidemiology of Campylobacter Infection. Clin Microbiol Rev.

[2] WHO. The global view of campylobacteriosis: report of an expert consultation: Report of an expert consultation, Utrecht, Netherlands, 9–11 July 2012. 2013.

[3] Man SM (2011). The clinical importance of emerging Campylobacter species. Nat Rev Gastroenterol Hepatol.

[4] Kapperud G, Skjerve E, Bean NH, Ostroff SM, Lassen J (1992). Risk factors for sporadic Campylobacter infections: results of a case-control study in southeastern Norway. J Clin Microbiol.

[5] Kapperud G, Lassen J, Ostroff SM, Aasen S (1992). Clinical features of sporadic Campylobacter infections in Norway. Scand J Infect Dis.

[6] Dionisi AM, Milito C, Martini H, Pesce AM, Mitrevski M, Granata G (2011). High prevalence of intestinal carriage of Campylobacter coli in patients with primary antibody deficiencies: a silent infection that could shift to a life-threatening condition. J Clin Gastroenterol.

[7] Melamed I, Bujanover Y, Igra YS, Schwartz D, Zakuth V, Spirer Z (1983). Campylobacter enteritis in normal and immunodeficient children. Am J Dis Child.

[8] Salyers WJ, Vega KJ, Munoz JC, Trotman BW, Tanev SS (2014). Neuroendocrine tumors of the gastrointestinal tract: case reports and literature review. World J Gastrointest Oncol.

[9] Puylaert JB, Vermeijden RJ, van der Werf SD, Doornbos L, Koumans RK (1989). Incidence and sonographic diagnosis of bacterial ileocaecitis masquerading as appendicitis. Lancet.

[10] van Spreeuwel JP, Lindeman J, Bax R, Elbers HJ, Sybrandy R, Meijer CJ (1987). Campylobacter-associated appendicitis: prevalence and clinicopathologic features. Pathol Annu.

[11] Blaser MJ (1997). Epidemiologic and clinical features of Campylobacter jejuni infections. J Infect Dis.

[12] Loss RW, Mangla JC, Pereira M (1980). Campylobacter colitis presentin as inflammatory bowel disease with segmental colonic ulcerations. Gastroenterology.

[13] Kunz PL (2015). Carcinoid and neuroendocrine tumors: building on success. J Clin Oncol.

